# Knowledge, attitudes and practice questionnaires in dengue, Zika, chikungunya and yellow fever settings: a scoping review protocol

**DOI:** 10.1136/bmjopen-2024-090251

**Published:** 2024-12-10

**Authors:** Claudia Ximena Robayo Gonzalez, Bouchra Nasri, Daniel Szaroz, Kate Zinszer

**Affiliations:** 1 École de Santé Publique, Département de Médecine Sociale et Préventive, Université de Montréal, Montréal, Québec, Canada; 2 Centre de Recherche en Santé Publique (CReSP), Montréal, Québec, Canada

**Keywords:** Surveys and Questionnaires, Knowledge, Public health, Epidemiology, Attitude, Behaviour

## Abstract

**Abstract:**

**Introduction:**

Arboviruses are a broad classification of viral pathogens that require vectors such as mosquitoes for infection transmission. The burden of arboviral diseases worldwide is substantial, affecting millions of people annually, with the *Aedes aegypti* mosquito responsible for spreading several common arboviruses, including dengue, chikungunya, Zika and yellow fever. One public health strategy to control and prevent these viruses is to influence community members’ behaviours related to reducing the breeding sites of *Aedes* mosquitoes, and knowledge, attitudes and practice (KAP) questionnaires are often used as part of these education campaigns.

**Objectives:**

To explore the content of KAP questionnaires and methodologies used to evaluate arboviral infections, focusing on dengue, Zika, chikungunya and yellow fever.

**Methods and analysis:**

To identify and describe KAP questionnaires for the selected arboviral infections, a scoping review will be performed and reported according to the Preferred Reporting Items for Systematic Reviews and Meta-Analyses Scoping Review Extension guidelines. Scientific databases such as MEDLINE, Cochrane, EMBASE, Web of Science, Scielo and LILACS will be searched systematically. Two independent reviewers will screen the title and abstract, followed by a full-text review of the selected articles using the COVIDENCE platform. The extracted information will include citation details, the type of arbovirus, the type of questions in each domain, the scoring system, the theoretical framework and the statistical analysis. The results will be presented comprehensively in tables and figures.

**Ethics and dissemination:**

Ethics approval is not required. Knowledge transfer will be conducted through conference presentations and publications.

**STRENGTHS AND LIMITATIONS OF THIS STUDY**

- This review will examine the content, statistical analysis and theoretical frameworks used in knowledge, attitudes and practice (KAP) arbovirus studies.
- The inclusion of three languages and seven databases will provide a diverse selection of articles.
- This scoping review explores studies using KAP in arboviral endemic locations worldwide.
- The review’s main limitation is the exclusion of grey literature, such as public health programmes that have used KAP questionnaires.

## Introduction

 Arboviruses are arthropodborne viruses that transmit infections, including dengue, yellow fever, Zika and chikungunya.^1^ These viruses are a major public health concern due to the increasing magnitude of arbovirus infections worldwide and the health consequences of infection.^1^,^4^ Their transmission is influenced by many factors, including migration, climate change and unplanned urbanisation^1 2^ that provide breeding sites for the vectors in high-risk areas.^1 3^ Dengue, Zika, chikungunya and yellow fever share the same vector and, therefore, the same control vector strategies for *Aedes* mosquitoes.^4^ The symptoms of infection can be similar among the four viruses, sharing symptoms such as fever, headache, joint pain, nausea, vomiting, rash and swollen glands.^5^ However, dengue can vary from asymptomatic to severe illness, and the severe form of dengue can be haemorrhagic, causing plasma leakage and organ failure with a fatality rate of 5%.^5^,^7^ With chikungunya infections, the primary symptom is joint pain, with chronic symptoms compromising neurological, cardiovascular and renal systems.^8^ For the Zika infection, the risk of congenital complications during pregnancy is estimated between 5% and 15%^9^ and can result in Guillian-Barré syndrome among those infected.^10^ Yellow fever also has a severe form that can be haemorrhagic and can lead to kidney and liver failure, which has a high fatality rate ranging between 30% and 60%.^11^

One public health strategy to control and prevent arbovirus infections is to influence community members’ behaviours related to reducing the breeding sites of *Aedes* mosquitoes.^12 13^ This can be achieved through involving community efforts to remove stagnant water, removing objects that collect stagnant water (eg, trash, discarded tires) and promoting environmental sanitation.^14^ To effectively engage and motivate community members, it is necessary to understand their attitudes towards these kinds of initiatives, their perception of social pressure related to practising or not practising certain behaviours (subjective norm), and their ability to carry out these actions (perceived behavioural control).^15^,^17^ This concept is known as the ‘Theory of Planned Behaviour framework’ and proposes how positive or negative experiences, social pressure and perceived control influence behaviour engagement.^15^ It considers the individual’s previous knowledge of the topic, the subjective norms’ influence over attitudes, and the individual’s perceived ability or confidence to engage in the practices.^15^,^17^ The use of knowledge, attitudes and practice (KAP) questionnaires is a way in which different elements of the theory of planned behaviour can be captured regarding a particular community intervention.^17^,^19^

KAP questionnaires are among the most common ways to evaluate behaviour change. The KAP model was developed in the 1950s to address difficulties in implementing family planning programmes in Africa.^1820^,^22^ KAP questionnaires have been used worldwide to study vectorborne diseases regarding knowledge about the disease, attitudes towards control and preventive practices.^23^,^28^ A typical objective of a KAP questionnaire is to evaluate what is known (knowledge), what the opinions (attitude) are, and what is done (practice) regarding a specific health problem in a community.^22 29^ Historically, studies often focused on evaluating a community’s knowledge of public health concepts related to public health programmes.^20^ Today, knowledge is used to examine information about a disease (and modes of transmission if it is an infectious disease) and related symptoms; attitudes refer to a person’s general feelings towards the disease and the different efforts or interventions that can be used to prevent and control the disease,^29 30^ and practice questions are considered preventive behaviours, if the individual or household is currently practising certain prevention efforts.^16^ Together, this information can be used to inform public health education campaigns,^28 31 32^ which requires an analysis of the KAP questionnaire data. Importantly, there is wide variation in how the ‘KAP’ outcome is created, including the scoring system of the different questions within each domain and between domains as well as the approaches used to create a KAP index.^24^,^2632 33^ Often, recommendations are not followed.^20 29 30 34^ In implementing and using KAP questionnaires, the analysis of the results does not always consider the recommendations regarding the scoring approach, how to consider relations between domains^29 30^ and the use of a behavioural theory to guide the approach.^17^,^1935 36^

In terms of arboviruses and KAP studies, two systematic reviews have evaluated different KAP questionnaire-based studies in the context of dengue in the Philippines and Malaysia, respectively.^37 38^ Additionally, there are four registered protocols for systematic reviews on KAP related to arboviruses. One protocol includes 15 arboviruses classified as mosquitoborne emerging infectious diseases that are global in scope.^39^ It will examine whether a health behaviour theory was used, whether it was used in specific parts of the questionnaire (development, analysis or discussion), and whether findings were contextualised to the setting.^39^ The second protocol considers dengue and KAP questionnaires based on studies conducted in Southeast Asia.^40^ The third protocol focuses on dengue in Latin American KAP questionnaire-based cross-sectional studies. It aims to describe the results of each domain and their relations to key determinant factors in the Latin American context.^41^ The last protocol, which focused on West Nile Virus, aims to conduct a comprehensive descriptive analysis of the general population’s knowledge level, attitudes towards the disease and prevention measures and protective behaviours.^42^ In addition to the differences noted above, none of the systematic reviews focuses or will focus on describing the scoring methods used in the individual KAP studies, with only one review considering the use of health behaviour theory in the included studies. Furthermore, all the reviews are based on articles written only in English and are restricted to certain regions of the world, with the exception of one review. Given the broad use of KAP questionnaires and the variability of arboviral infections over time and between contexts,^29 43 44^ a comprehensive approach is essential to gain insights into the content and use of these questionnaires in different temporal and contextual settings. By consolidating and analysing the information in the literature, this scoping review aims to describe the current state of KAP-based studies, evolving trends and emerging needs in KAP assessment methodologies.

### Objectives

This scoping review aims to explore the content of KAP questionnaires and methodologies used to evaluate arboviral studies focusing on dengue, Zika, chikungunya and yellow fever. The specific objectives are as follows:

- Provide a comprehensive description of the content in KAP questionnaires used in health research.
- Analyse the various methodologies employed in assessing KAP, including indices, separate indicators for each domain and other relevant methods.
- Identify the analysis used to evaluate the relationship between the domains.
- Examine and describe the construction of KAP scores and how these scores were used in analysing the results.
- Evaluate the incorporation and use of theoretical frameworks to guide the design and/or analysis of the KAP questionnaire.

## Methods and analysis

The Preferred Reporting Items for Systematic Reviews and Meta-Analyses (PRISMA) Scoping Review Extension guidelines checklist will be followed.^45^ The search equations will be adapted to librarian recommendations. The protocol is registered in the Open Science Framework (osf.io) and accessed at https://osf.io/cvw4q.

The scoping review follows the methodology developed by Arksey and O’Malley,^46^ which comprises five stages: (1) defining the research questions, (2) identifying relevant studies, (3) selecting eligible studies, (4) organising the data and (5) summarising, combining and reporting the results. Procedures such as the literature search, data extraction and synthesis of the findings are planned between November 2024 and 2025.

### Stage 1: identifying the research questions

Given the nature of the KAP questionnaires and the different domains that must be considered in the questionnaire and the analysis, a main question and four additional questions will help guide the data extraction. These are as follows:

How are the domains in KAP questionnaires on the selected arboviruses developed and organised?

1. What methods were used to analyse the data captured by the KAP questionnaires?
2. How were the relations between the domains studied?
3. What scoring approach to KAP was used, if any?
4. Was there any use of a theoretical framework for behavioural change in constructing the questionnaire and/or guiding the analysis?

### Stage 2: identifying relevant studies

From 1980 until today, the number of scientific publications regarding these four arboviruses has increased steadily. In 1990, only 75 publications involving the Caribbean region were related to dengue; in 2020, that number increased to 1053.^47^ As for chikungunya and Zika-related publications, an increase was reported after 2014 and 2016, respectively.^47^ Thus, the search will include studies from 2000 to the present, accounting for the increase in publications on the four arboviruses along the time frame (2000–2024) and the increase in KAP studies published after this year. The terms and strategies that will be used are presented in table 1. The search strategy will be applied to databases such as Cochrane, Medline, Web of Science, EMBASE and SCOPUS using key terms selected from MEDLINE Medical Subject Headings (MeSH). For Scielo and LILACS, the Descriptores en Ciencias de la Salud or DeCS will be used. Both will be combined with Boolean and proximity operators (online supplemental material 1).

**Table 1:** Concepts and MeSH/DeCS terms

English	Spanish
Knowledge, attitudes and practice Knowledge, attitudes and practice Knowledge, attitudes, practice and behaviour KAP surveys KAP KAPB surveys KAP questionnaire Health knowledge attitudes practice Knowledge* Attitude to health Public health practice* health behaviour*	Conocimientos, actitudes y practicas. Conocimientos, Actitudes y Práctica en Salud CAP Conocimientos, Actitudes y Práctica Sanitarias Conocimientos, Actitudes y Prácticas en Salud Encuestas CAP Encuestas de conocimientos, actitudes y prácticas Conocimientos en salud Prácticas en salud Actitud hacia la prevención
Arbovirus Arbovirus Dengue infection DENV serotype Dengue Severe dengue Zika virus infection Zika infection Yellow fever Yellow fever infection Chikungunya infection Chikungunya fever arthropodborne virus break bone fever Classical dengue Classical dengue fever Classical dengue fevers Classical dengues	Arbovirus Infecciones por Arbovirus Arbovirus Dengue Dengue Grave Virus del dengue Virus Zika Infección por el zika virus Fiebre amarilla Virus de la fiebre amarilla Virus Chikunguña Fiebre Chikunguña Virus Transmitido por Artrópodos Virus Transmitidos por Artrópodos

* Not MeSH terms added to the search equation.

CAPconocimientos, actitudes y practicasKAPknowledge, attitudes and practiceKAPBknowledge, attitudes, practice and behaviour

The inclusion criteria are studies that used KAP questionnaires to collect data on selected arboviruses such as dengue, Zika, chikungunya and yellow fever. The studies must be written in English, French or Spanish, from endemic locations according to WHO and Centers for Disease Control and Prevention (CDC).^9 48 49^ We will exclude commentaries, editorials, media reviews, opinion pieces, as well as questionnaires implemented solely on healthcare workers, students and other professionals. Additionally, articles focusing solely on developing and validating questionnaires, those lacking the complete questionnaire, or not evaluating all three domains will be excluded. Furthermore, we will not consider systematic reviews, meta-analyses or grey literature. Refer to table 2 for more details on the inclusion and exclusion criteria.

**Table 2:** Inclusion and exclusion criteria.

	Inclusion criteria	Exclusion criteria
Population	The general population or communities in locations that are endemic to the selected arboviruses	Non-endemic locations for the selected arbovirus infectionsHealthcare workers, higher academic institutions, military personnel, construction workers, agricultural workers, prisons and nursing homes
Concept	Results and analysis of the implementation of KAP or KAPB questionnairesArticles providing the complete questionnaire	Articles presenting the KAP/KAPB questionnaire development or validation process and not the results of administering the questionnaire on a selected populationArticles evaluating and analysing one or two of the three domains and not all three domainsArticles where the questionnaire is not available
Context	Arboviruses that are transmitted by Aedes mosquitoes. (dengue, Zika, chikungunya, yellow fever)	Arboviruses including West Nile Fever, Japanese Encephalitis, Powassan virus, California encephalitis, Eastern Equine Encephalomyelitis, St. Louis Encephalitis and Venezuelan Equine Encephalitis Virus
Sources	Journal articles, peer-reviewed, original researchFull-text articles	Books, book chapters, editorials, erratum, opinion pieces, conference abstracts, dissertations, systematic reviews, meta-syntheses and analyses, and commentaries
Focus	Randomised trials, observational studies (cross-sectional, case-control, cohort)	Grey literaturePublic health programmes that used KAP/KAPB
Language	English, French, Spanish	Other languages
Year	2000–2024	Before 2000

KAPknowledge, attitudes and practiceKAPBknowledge, attitudes, practice and behaviour

### Stage 3: selecting eligible studies

**Figure 1:**
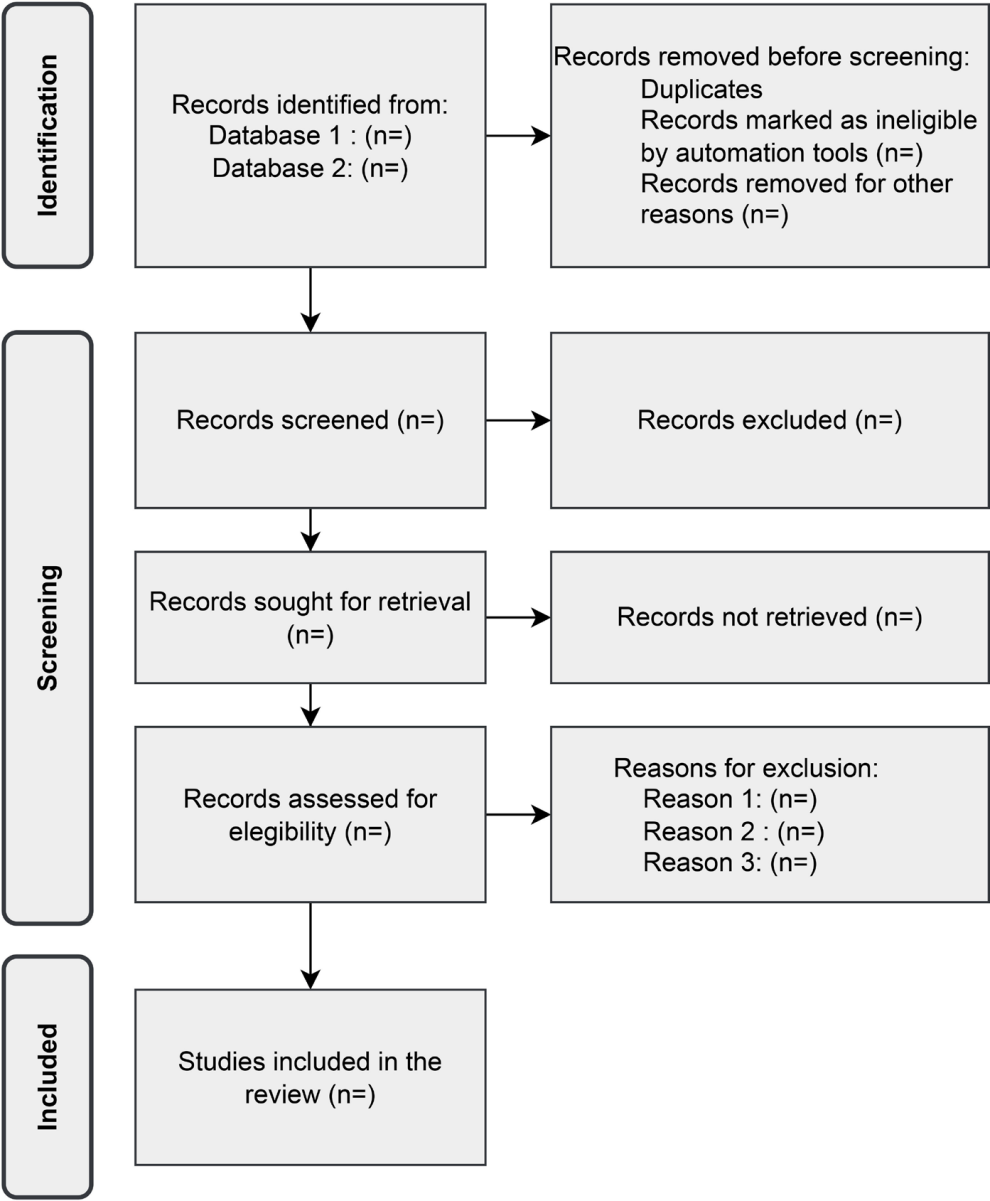
Preferred Reporting Items for Systematic Reviews and Meta-Analyses extension for Scoping Reviews flow diagram for study selection.^55^

The results from the different databases will be uploaded to COVIDENCE.^50^ After removing duplicates, two independent reviewers, PhD students (CXRG and DS), will evaluate the articles. In the first stage of the review, article titles and abstracts will be assessed based on the inclusion and exclusion criteria. In the second stage, the included papers will be evaluated by two reviewers. The inclusion of articles for the extraction phase will be based on consensus. Their inter-rater reliability will be qualitatively evaluated at this phase. Disagreements will be resolved through discussion, and a third senior reviewer (KZ) will be involved in case of persistence. The reasons for exclusion in this phase will be recorded for each study. A PRISMA flow chart will be presented to summarise the inclusion and exclusion of articles (figure 1).

### Stage 4: charting the data

Data will be extracted using a pilot-tested form. These data include the author of the study, the year of publication, the study objectives, the targeted study population, the geographical location, the specific type of arbovirus being analysed, the various KAP questions categorised by domain, the type of questions used for each domain (such as multiple options, Likert scale, true or false), a thorough description of the scoring system employed, whether or not a theoretical framework was used for the analysis, the results obtained, the statistical analysis conducted and the conclusions drawn from the study regarding the associations/correlations between among the domains (table 3). The Cochrane RoB 2^51^ will be used for randomised controlled trials, and the Newcastle-Ottawa scale will be used to evaluate the quality of observational studies.^52^ Two reviewers will extract data independently.

**Table 3:** Preliminary data extraction form

Element	Description
Title	The title of the articles
Author	List the author(s)
Year	Year of publication
Aims/purpose	Objective of the study
Research design	RCT, cohort, cross-sectional, etc
Region	According to the WHO regions, Americas, Asia and Africa
Country	Country in which the study was conducted
Population	Community members, mothers, fathers, caretakers, adolescents
Type of arbovirus	Dengue, Zika, chikunguya and yellow fever
Number of questions in the questionnaire	The number of questions in the questionnaire, including sociodemographics
Number of questions in Knowledge	The number of questions
Number of questions in attitudes	The number of questions
Number of questions in practice/behaviour	The number of questions
Type of questions in knowledge	Multiple options, Likert scale, true or false
Type of questions in attitudes	Multiple options, Likert scale, true or false
Type of questions in practice/behaviour	Multiple options, Likert scale, true or false
Scoring system	Yes or no
Scoring system by domain or total	By domain, total or both
Description of the scoring system	Description of the use or construction of a scoring system
Use of theoretical framework in the development of the questionnaire	Yes or no
Use of theoretical framework in the analysis	Yes or no
Theoretical framework used	Name of the framework
Evaluation of the relation between domains	Yes or no
Type of analysis used to evaluate the relation between domains	Name of the analysis
KAP statistical analysis conducted	Name of the analysis
Principal results from the statistical analysis	Present the measure and the analysis made by the authors

RCTrandomised controlled trial

### Stage 5: collating, summarising and reporting the results

The collected data will undergo a rigorous analysis process consistent with established guidelines for the development and analysis of KAP questionnaires.^20 53 54^ This analysis will comprehensively examine the data, including categorising key findings of the relations between the domains and assessing methodological approaches employed across studies. Descriptive statistical analysis will be employed to summarise the characteristics of the identified literature. The results will be presented clearly and concisely using tables, figures and narrative descriptions to illustrate the breadth and depth of the literature. Additionally, a discussion will provide contextual explanations and elucidate the implications of the findings within the broader field of arbovirus infections and public health. Throughout this process, similarities and differences in the content of the different KAP questionnaires and less explored areas in the questionnaires will be identified. Moreover, identifying the methodologies employed for developing the scoring systems and the overall assessment of the KAP will show how to create these scores, the complexity they have, and the strengths and weaknesses to be improved in future studies. Regarding the relationship between the domains, the results will present evidence on how these relationships have been evaluated, highlighting the novel analytical methods employed. Finally, identifying a theoretical behavioural framework in the analyses will provide insight into one of the most widely used, the impact on the final analysis, and how these theoretical frameworks contribute to understanding KAP dynamics. The scoping review seeks to thoroughly examine and summarise the existing research on KAP questionnaires in the context of arbovirus infections. The review aims to provide in-depth insights into this area of study and to offer valuable recommendations for the future use, development and analysis of KAP questionnaires.

### Patient and public involvement

There was no involvement of patients or the public in the protocol’s design.

## Ethics and dissemination

This review does not require ethical approval as it involves the secondary analysis of existing data from publicly available sources. The findings will be disseminated through publication in appropriate peer-reviewed journals and presentations at relevant conferences.

## Limitations

Conducting a scoping review is challenging, particularly when assessing KAP questionnaires. One of the major challenges is the variability in the questionnaires and the different approaches used in the analyses, including the scoring system. Another limitation is the inclusion of only studies with available questionnaires for scoping review, leaving out some articles that could have other inclusion criteria. Additionally, language restrictions may limit the availability of articles from regions where certain diseases or health conditions are endemic, making it difficult to obtain a comprehensive overview.

## supplementary material

10.1136/bmjopen-2024-090251 online supplemental file 1
