# Supplementary material for: Knowledge, attitudes and practice questionnaires in dengue, Zika, chikungunya and yellow fever settings: a scoping review protocol
Source: BMJ Open. 2024 Dec 10;14(12):e090251. doi: 10.1136/bmjopen-2024-090251 (PMC11647391; doi:10.1136/bmjopen-2024-090251)
Supplement: online supplemental file 1 [file bmjopen-14-12-s001.docx]

**Medline**

| Concept 1  KAP questionnaires | 1 exp Attitude to Health/  2 public health practice/ or exp population surveillance/ or exp primary prevention/  3 health behavior/ or health risk behaviors/  4 ((knowledge* or attitude* or practice* or KAP or KAPB) adj2 (health or survey* or questionnaire*)).ti,ab,kf.  5 or/1-4 [KAP] |
| --- | --- |
| Concept 2  Arboviruses | 6 Arboviruses/  7 exp Dengue/ or Dengue Virus/  8 Zika Virus/  9 Yellow Fever/  10 Yellow fever virus/  11 Zika Virus Infection/  12 Chikungunya Fever/ or Chikungunya virus/  13 ((Dengue or "yellow fever" or chikungunya or zika or DENV or arbovir* or "arthropod borne") adj2 (infection* or serotype* or virus* or fever)).ti,ab,kf.  14 or/6-13 [arbovirus]  15 5 and 14  16 limit 15 to (yr="2000 -Current" and (english or french or spanish)) |

**SCIELO**

| Concept 1  KAP questionnaires | (ab:((ab:(Conocimientos, Actitudes y Práctica en Salud)))) OR (ab:("CAP")) OR (ab:(Conocimientos, Actitudes y Práctica Sanitarias)) OR (ab:(Conocimientos, Actitudes y Prácticas en Salud)) OR (ab:(Encuestas CAP)) OR (ab:(Encuestas de conocimientos, actitudes y prácticas )) OR (ab:(Conocimientos en salud)) OR (ab:(Prácticas en salud)) OR (ab:(Actitud hacia la prevención)) |
| --- | --- |
| Concept 2  Arboviruses | (ab:(Infecciones por Arbovirus)) OR (ab:(Arbovirus)) OR (ab:(Dengue )) OR (ab:(Dengue Grave)) OR (ab:(Virus del dengue)) OR (ab:(Virus Zika)) OR (ab:(Infección por el zika virus)) OR (ab:(Fiebre amarilla)) OR (ab:(Virus de la fiebre amarilla)) OR (ab:(Virus Chikunguña)) OR (ab:(Fiebre Chikunguña)) OR (ab:(Virus Transmitido por Artrópodos)) OR (ab:(Virus Transmitidos por Artrópodos)) |
