# Supplementary material for: Knowledge, attitudes and practice questionnaires in dengue, Zika, chikungunya and yellow fever settings: a scoping review protocol
Source: BMJ Open. 2024 Dec 10;14(12):e090251. doi: 10.1136/bmjopen-2024-090251 (PMC11647391; doi:10.1136/bmjopen-2024-090251)
Supplement: Supplementary file 2 [file bmjopen-2024-090251.reviewer_comments.pdf]

PEER REVIEW HISTORY

BMJ Open publishes all reviews undertaken for accepted manuscripts. Reviewers are asked to complete a checklist review form and are provided with free text boxes to elaborate on their assessment. These free text comments are reproduced below.

ARTICLE DETAILS

Title (Provisional)

Knowledge attitudes and practice questionnaires in dengue, Zika, chikungunya and yellow fever settings: A Scoping Review Protocol.

Authors

Robayo Gonzalez, Claudia Ximena; Nasri, Bouchra; Szaroz, Daniel; Zinszer, Kate

---

VERSION 1 - REVIEW

**Reviewer**	**1**
**Name**	**SAHIMIN, NORHIDAYU**
**Affiliation**	**Universiti Malaya, Kuala Lumpur**
**Date**	**22-Aug-2024**
**COI**	**There is no competing interest.**

The protocol was good—minor comments were provided in the attachment.

****The reviewer provided a marked copy with additional comments. Please contact the publisher for full details.**

**Reviewer**	**2**
**Name**	**Dittrich, Sabine**
**Affiliation**	**FIND**
**Date**	**05-Oct-2024**
**COI**	**No COIs**

This is a very nice piece of work and the resulting study will be very useful to help inform disease interventions based on people's knowledge and behavior. One thing to consider is to potentially include search terms related to "vector or mosquito-borne diseases" to ensure not missing any papers.

VERSION 1 - AUTHOR RESPONSE

1. Reviewer 1: How do you determine endemic/ non-endemic countries? Any references?

A. The list of endemic countries was found on the World Health Organization (WHO) and the Centers for Disease Control (CDC) web page from each disease. This has been clarified in the manuscript and a reference added on page 8.

2. Reviewer 1: How do you plan to overcome the scoring system problem?

A. The data extraction process will detail the methodologies used to develop the scores, the grouping of the questions, and the standardization of the scores. The description is in the subsection stage 5, on page 11.

3. Reviewer 2: One thing to consider is to potentially include search terms related to "vector or mosquito-borne diseases" to ensure not missing any papers.

A. Thank you for the suggestion. We reviewed our initial search strategy and can confirm that mosquito-borne disease is included in the keywords for each of the viruses as well as the keyword “arthropod.” For example, the main term for dengue includes mosquito-borne disease. The same happens with the other three diseases. We did not include vector-borne disease as a term as this would have unnecessarily widened the search to include several diseases that we are not focused on.

I hope this clarifies the information. Thank you for your comments, which help to improve the manuscript and the protocol.

I appreciate your consideration.
